# 2-[(2-Hy­droxy­eth­yl)aza­nium­yl]ethanaminium oxalate monohydrate

**DOI:** 10.1107/S1600536811056157

**Published:** 2012-01-11

**Authors:** Yu Jin

**Affiliations:** a Ordered Matter Science Research Center, Southeast University, Nanjing 211189, People’s Republic of China

## Abstract

In the title hydrated mol­ecular salt, C_4_H_14_N_2_O^2+^·C_2_O_4_^2−^·H_2_O, the oxalate dianion is almost planar (r.m.s. deviation = 0.020 Å). In the crystal, the components are linked by N—H⋯O(water), N—H⋯O(oxalate) O—H(ammonium)⋯O(oxalate), O—H(water)⋯O(oxalate) and O—H(water)⋯O(ammonium) hydrogen bonds, thereby forming a complex three-dimensional packing motif.

## Related literature

For related structures, see: Sakai *et al.* (2003 ▶); Kolitsch (2004 ▶); Cotton *et al.* (1996 ▶); Barnes (2003 ▶).
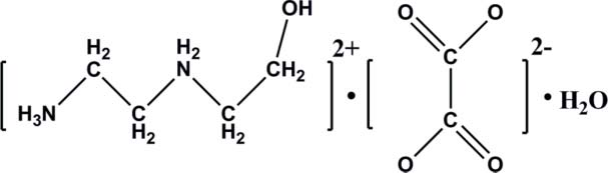


## Experimental

### 

#### Crystal data

C_4_H_14_N_2_O^2+^·C_2_O_4_^2−^·H_2_O
*M* *_r_* = 212.21
Monoclinic, 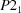

*a* = 5.7311 (11) Å
*b* = 13.136 (3) Å
*c* = 6.7373 (13) Å
β = 102.52 (3)°
*V* = 495.16 (17) Å^3^
*Z* = 2
Mo *K*α radiation
μ = 0.13 mm^−1^
*T* = 293 K
0.3 × 0.3 × 0.2 mm

#### Data collection

Rigaku Mercury CCD diffractometer
Absorption correction: multi-scan (*CrystalClear*; Rigaku, 2005 ▶) *T*_min_ = 0.489, *T*_max_ = 1.000
5068 measured reflections
2261 independent reflections
1853 reflections with *I* > 2σ(*I*)
*R*_int_ = 0.046

#### Refinement

*R*[*F* ^2^ > 2σ(*F* ^2^)] = 0.038
*wR*(*F* ^2^) = 0.086
*S* = 0.97
2261 reflections
135 parameters
3 restraints
H atoms treated by a mixture of independent and constrained refinement
Δρ_max_ = 0.22 e Å^−3^
Δρ_min_ = −0.25 e Å^−3^

### 

Data collection: *CrystalClear* (Rigaku, 2005 ▶); cell refinement: *CrystalClear*; data reduction: *CrystalClear*; program(s) used to solve structure: *SHELXS97* (Sheldrick, 2008 ▶); program(s) used to refine structure: *SHELXL97* (Sheldrick, 2008 ▶); molecular graphics: *SHELXTL* (Sheldrick, 2008 ▶); software used to prepare material for publication: *SHELXL97*.

## Supplementary Material

Crystal structure: contains datablock(s) I, global. DOI: 10.1107/S1600536811056157/hb6584sup1.cif

Structure factors: contains datablock(s) I. DOI: 10.1107/S1600536811056157/hb6584Isup2.hkl

Supplementary material file. DOI: 10.1107/S1600536811056157/hb6584Isup3.cml

Additional supplementary materials:  crystallographic information; 3D view; checkCIF report

## Figures and Tables

**Table 1:** Hydrogen-bond geometry (Å, °)

*D*—H⋯*A*	*D*—H	H⋯*A*	*D*⋯*A*	*D*—H⋯*A*
N1—H1*A*⋯O1*W*^i^	0.89	1.96	2.823 (2)	164
N1—H1*B*⋯O3^ii^	0.89	2.12	2.8769 (19)	143
N1—H1*B*⋯O4^ii^	0.89	2.11	2.818 (2)	136
N1—H1*F*⋯O2	0.89	1.82	2.707 (2)	172
N2—H2*A*⋯O4^iii^	0.90	1.80	2.688 (2)	170
N2—H2*D*⋯O5^iv^	0.90	2.16	2.862 (2)	134
N2—H2*D*⋯O2^iv^	0.90	2.00	2.773 (2)	143
O1—H1*C*⋯O3^v^	0.82	1.94	2.736 (2)	163
O1*W*—H2*W*⋯O5^iv^	0.84 (1)	1.91 (1)	2.753 (2)	178 (2)
O1*W*—H1*W*⋯O1^vi^	0.84 (1)	2.27 (3)	2.968 (2)	141 (4)

Symmetry codes: (i) 
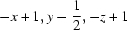
; (ii) 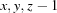; (iii) 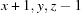; (iv) 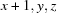; (v) 
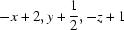
; (vi) 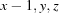.

**Fig. 1.**
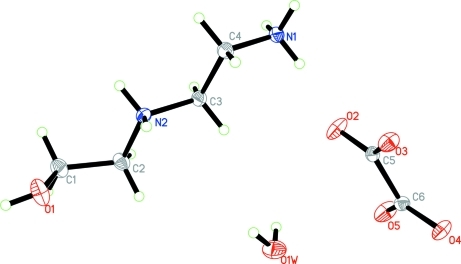
Molecular structure of the title compound with displacement ellipsoids drawn at the 30% probability level.

**Fig. 2.**
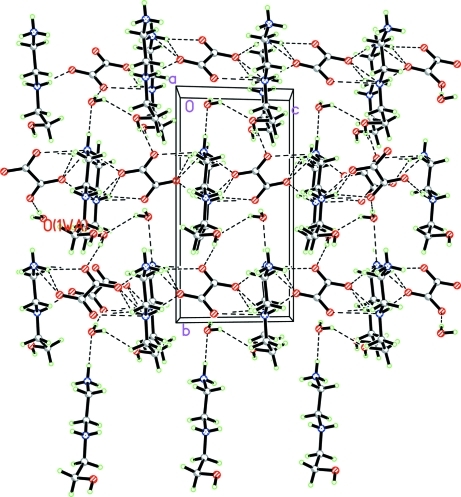
Crystal structure of the title compound with view along the a axis. Intermolecular interactions are shown as dashed lines.

## supplementary crystallographic information

### Comment

Several crystal structures of oxalate have been reported previously (Sakai *et al.*, 2003; Kolitsch, 2004; Cotton *et al.*, 1996). As an extension of research, we report here the synthesis and the crystal structure of the title complex, (C_4_H_14_N_2_O)^2+^.(C_2_O_4_)^2-^.H_2_O.

In the crystal synthesized by Barnes, amine salts with oxalic acid contain the monohydrogenoxalate ion (Barnes, 2003), while the crystal reported here, oxalic acid reacts with alcohol amine to give crystals of the fully deprotonated C_2_O_4_^2-^ salt as the monohydrate.

The (locally) centrosymmetric anion and one cation are shown in Fig. 1 with the hydrogen bonds listed in Table 1. The water molecules in the compound serve as a connection, *i.e.*, two protonated cations are connected to a water molecule through N—H···O (water) and O—H (water)···O (ammonium) hydrogen-bonds and one anion is linked to the same water molecule *via* O—H (water)···O (oxalate) hydrogen bonding interactions, the components are further held by O—H (ammonium)···O (oxalate), O—H (water)···O (oxalate) and O—H (water)···O (ammonium) hydrogen-bonding interactions, and thus forms a three-dimensional structure. (Fig.2)

### Experimental

A mixture of C_4_H_12_N_2_O (104.15 mg, 1.00 mmol), C_2_H_2_O_4_ (90.04 mg, 1.00 mmol) and distilled water (5 ml) was stirred a few minutes at room temperature, giving a clear transparent solution. After evaporation for several days, colorless blocks of the title compound were obtained in about 82% yield and filtered and washed with distilled water.

### Refinement

The absolute sturcture is indeterminate based on the present refinement. H atoms bound to carbon and nitrogen were placed at idealized positions [C—H = 0.97 Å, O—H = 0.82 to 0.84 Å and N—H = 0.89 to 0.90 Å] and allowed to ride on their parent atoms with *U*_iso_ fixed at 1.2 *U*_eq_(C,*N*).

### Crystal data

**Table:**

C_4_H_14_N_2_O^2+^·C_2_O_4_^2^^−^·H_2_O	*F*(000) = 228
*M**_r_* = 212.21	*D*_x_ = 1.423 Mg m^−^^3^
Monoclinic, *P*2_1_	Mo *K*α radiation, λ = 0.71073 Å
Hall symbol: P 2yb	Cell parameters from 3450 reflections
*a* = 5.7311 (11) Å	θ = 6.2–55.3°
*b* = 13.136 (3) Å	µ = 0.13 mm^−^^1^
*c* = 6.7373 (13) Å	*T* = 293 K
β = 102.52 (3)°	Block, colorless
*V* = 495.16 (17) Å^3^	0.3 × 0.3 × 0.2 mm
*Z* = 2	

### Data collection

**Table:**

Rigaku Mercury CCD diffractometer	2261 independent reflections
Radiation source: fine-focus sealed tube	1853 reflections with *I* > 2σ(*I*)
graphite	*R*_int_ = 0.046
ω scans	θ_max_ = 27.5°, θ_min_ = 3.1°
Absorption correction: multi-scan (*CrystalClear*; Rigaku, 2005)	*h* = −7→7
*T*_min_ = 0.489, *T*_max_ = 1.000	*k* = −16→16
5068 measured reflections	*l* = −8→8

### Refinement

**Table:**

Refinement on *F*^2^	Primary atom site location: structure-invariant direct methods
Least-squares matrix: full	Secondary atom site location: difference Fourier map
*R*[*F*^2^ > 2σ(*F*^2^)] = 0.038	Hydrogen site location: inferred from neighbouring sites
*wR*(*F*^2^) = 0.086	H atoms treated by a mixture of independent and constrained refinement
*S* = 0.97	*w* = 1/[σ^2^(*F*_o_^2^) + (0.0314*P*)^2^] where *P* = (*F*_o_^2^ + 2*F*_c_^2^)/3
2261 reflections	(Δ/σ)_max_ < 0.001
135 parameters	Δρ_max_ = 0.22 e Å^−^^3^
3 restraints	Δρ_min_ = −0.25 e Å^−^^3^

### Special details

**Table:**

Geometry. All e.s.d.'s (except the e.s.d. in the dihedral angle between two l.s. planes) are estimated using the full covariance matrix. The cell e.s.d.'s are taken into account individually in the estimation of e.s.d.'s in distances, angles and torsion angles; correlations between e.s.d.'s in cell parameters are only used when they are defined by crystal symmetry. An approximate (isotropic) treatment of cell e.s.d.'s is used for estimating e.s.d.'s involving l.s. planes.
Refinement. Refinement of *F*^2^ against ALL reflections. The weighted *R*-factor *wR* and goodness of fit *S* are based on *F*^2^, conventional *R*-factors *R* are based on *F*, with *F* set to zero for negative *F*^2^. The threshold expression of *F*^2^ > σ(*F*^2^) is used only for calculating *R*-factors(gt) *etc*. and is not relevant to the choice of reflections for refinement. *R*-factors based on *F*^2^ are statistically about twice as large as those based on *F*, and *R*- factors based on ALL data will be even larger.

### Fractional atomic coordinates and isotropic or equivalent isotropic displacement parameters (Å^2^)

**Table:**

	*x*	*y*	*z*	*U*_iso_*/*U*_eq_	
C1	1.1138 (4)	0.63105 (16)	0.1821 (3)	0.0342 (5)	
H1D	1.1520	0.5976	0.0648	0.041*	
H1E	1.0764	0.7017	0.1467	0.041*	
C2	0.9023 (4)	0.58091 (12)	0.2350 (3)	0.0290 (4)	
H2B	0.8808	0.6071	0.3644	0.035*	
H2C	0.7607	0.5980	0.1328	0.035*	
C3	0.6935 (4)	0.41905 (13)	0.2318 (3)	0.0256 (4)	
H3A	0.5915	0.4347	0.1007	0.031*	
H3B	0.6171	0.4452	0.3365	0.031*	
C4	0.7217 (4)	0.30554 (15)	0.2545 (3)	0.0284 (5)	
H4A	0.8062	0.2791	0.1555	0.034*	
H4B	0.8127	0.2888	0.3895	0.034*	
C5	0.2891 (3)	0.33640 (13)	0.6829 (2)	0.0213 (4)	
C6	0.1213 (3)	0.39951 (13)	0.7871 (3)	0.0234 (4)	
H1W	0.472 (5)	0.541 (3)	0.636 (3)	0.117 (14)*	
H2W	0.702 (3)	0.522 (2)	0.723 (3)	0.057 (9)*	
N1	0.4808 (3)	0.26039 (11)	0.2219 (2)	0.0244 (4)	
H1A	0.4931	0.1931	0.2351	0.037*	
H1B	0.3992	0.2758	0.0975	0.037*	
H1F	0.4048	0.2851	0.3134	0.037*	
N2	0.9269 (3)	0.46896 (11)	0.2484 (2)	0.0210 (3)	
H2A	0.9948	0.4465	0.1480	0.025*	
H2D	1.0235	0.4521	0.3678	0.025*	
O1	1.3118 (3)	0.62633 (11)	0.3457 (2)	0.0453 (4)	
H1C	1.4124	0.6679	0.3285	0.068*	
O2	0.2737 (3)	0.35255 (12)	0.49926 (18)	0.0391 (4)	
O3	0.4216 (3)	0.27372 (10)	0.78762 (18)	0.0320 (3)	
O4	0.1307 (3)	0.38042 (10)	0.96855 (18)	0.0333 (3)	
O5	−0.0084 (3)	0.46250 (12)	0.6815 (2)	0.0429 (4)	
O1W	0.5752 (3)	0.54801 (12)	0.7448 (2)	0.0425 (4)	

### Atomic displacement parameters (Å^2^)

**Table:**

	*U*^11^	*U*^22^	*U*^33^	*U*^12^	*U*^13^	*U*^23^
C1	0.0382 (13)	0.0278 (9)	0.0369 (10)	−0.0049 (9)	0.0084 (9)	0.0046 (8)
C2	0.0263 (11)	0.0223 (9)	0.0374 (10)	0.0008 (8)	0.0044 (8)	0.0020 (8)
C3	0.0216 (11)	0.0251 (9)	0.0314 (10)	−0.0002 (8)	0.0081 (8)	−0.0009 (7)
C4	0.0241 (11)	0.0250 (9)	0.0375 (11)	0.0007 (9)	0.0102 (9)	−0.0023 (8)
C5	0.0188 (10)	0.0222 (8)	0.0227 (8)	−0.0017 (7)	0.0045 (7)	0.0004 (7)
C6	0.0231 (11)	0.0248 (9)	0.0220 (8)	−0.0004 (8)	0.0039 (8)	−0.0028 (7)
N1	0.0279 (10)	0.0218 (7)	0.0242 (7)	−0.0011 (7)	0.0071 (7)	0.0015 (6)
N2	0.0213 (9)	0.0226 (7)	0.0192 (7)	0.0018 (7)	0.0047 (6)	0.0004 (6)
O1	0.0344 (9)	0.0474 (9)	0.0505 (9)	−0.0138 (8)	0.0015 (7)	0.0129 (7)
O2	0.0393 (10)	0.0576 (10)	0.0234 (7)	0.0198 (8)	0.0138 (6)	0.0067 (6)
O3	0.0358 (9)	0.0320 (7)	0.0290 (7)	0.0148 (7)	0.0086 (6)	0.0043 (5)
O4	0.0371 (9)	0.0424 (8)	0.0234 (6)	0.0127 (7)	0.0128 (6)	0.0031 (6)
O5	0.0495 (11)	0.0490 (8)	0.0305 (7)	0.0281 (8)	0.0091 (7)	0.0080 (7)
O1W	0.0448 (11)	0.0345 (8)	0.0478 (10)	0.0076 (8)	0.0091 (8)	−0.0079 (7)

### Geometric parameters (Å, °)

**Table:**

C1—O1	1.402 (2)	C5—O3	1.233 (2)
C1—C2	1.489 (3)	C5—O2	1.239 (2)
C1—H1D	0.9700	C5—C6	1.548 (3)
C1—H1E	0.9700	C6—O5	1.230 (2)
C2—N2	1.478 (2)	C6—O4	1.238 (2)
C2—H2B	0.9700	N1—H1A	0.8900
C2—H2C	0.9700	N1—H1B	0.8900
C3—N2	1.472 (2)	N1—H1F	0.8900
C3—C4	1.504 (3)	N2—H2A	0.9000
C3—H3A	0.9700	N2—H2D	0.9000
C3—H3B	0.9700	O1—H1C	0.8200
C4—N1	1.475 (3)	O1W—H1W	0.838 (10)
C4—H4A	0.9700	O1W—H2W	0.841 (10)
C4—H4B	0.9700		
			
O1—C1—C2	110.75 (16)	C3—C4—H4B	110.1
O1—C1—H1D	109.5	H4A—C4—H4B	108.4
C2—C1—H1D	109.5	O3—C5—O2	125.95 (17)
O1—C1—H1E	109.5	O3—C5—C6	117.66 (14)
C2—C1—H1E	109.5	O2—C5—C6	116.37 (15)
H1D—C1—H1E	108.1	O5—C6—O4	126.77 (19)
N2—C2—C1	112.53 (17)	O5—C6—C5	117.06 (15)
N2—C2—H2B	109.1	O4—C6—C5	116.18 (14)
C1—C2—H2B	109.1	C4—N1—H1A	109.5
N2—C2—H2C	109.1	C4—N1—H1B	109.5
C1—C2—H2C	109.1	H1A—N1—H1B	109.5
H2B—C2—H2C	107.8	C4—N1—H1F	109.5
N2—C3—C4	110.98 (15)	H1A—N1—H1F	109.5
N2—C3—H3A	109.4	H1B—N1—H1F	109.5
C4—C3—H3A	109.4	C3—N2—C2	111.45 (14)
N2—C3—H3B	109.4	C3—N2—H2A	109.3
C4—C3—H3B	109.4	C2—N2—H2A	109.3
H3A—C3—H3B	108.0	C3—N2—H2D	109.3
N1—C4—C3	107.88 (16)	C2—N2—H2D	109.3
N1—C4—H4A	110.1	H2A—N2—H2D	108.0
C3—C4—H4A	110.1	C1—O1—H1C	109.5
N1—C4—H4B	110.1	H1W—O1W—H2W	106 (3)

### Hydrogen-bond geometry (Å, °)

**Table:**

*D*—H···*A*	*D*—H	H···*A*	*D*···*A*	*D*—H···*A*
N1—H1A···O1W^i^	0.89	1.96	2.823 (2)	164
N1—H1B···O3^ii^	0.89	2.12	2.8769 (19)	143
N1—H1B···O4^ii^	0.89	2.11	2.818 (2)	136
N1—H1F···O2	0.89	1.82	2.707 (2)	172
N2—H2A···O4^iii^	0.90	1.80	2.688 (2)	170
N2—H2D···O5^iv^	0.90	2.16	2.862 (2)	134
N2—H2D···O2^iv^	0.90	2.00	2.773 (2)	143
O1—H1C···O3^v^	0.82	1.94	2.736 (2)	163
O1W—H2W···O5^iv^	0.84 (1)	1.91 (1)	2.753 (2)	178 (2)
O1W—H1W···O1^vi^	0.84 (1)	2.27 (3)	2.968 (2)	141 (4)

Symmetry codes: (i) −*x*+1, *y*−1/2, −*z*+1; (ii) *x*, *y*, *z*−1; (iii) *x*+1, *y*, *z*−1; (iv) *x*+1, *y*, *z*; (v) −*x*+2, *y*+1/2, −*z*+1; (vi) *x*−1, *y*, *z*.
